# Familial testicular germ cell tumor: no associated syndromic pattern identified

**DOI:** 10.1186/1897-4287-12-3

**Published:** 2014-02-21

**Authors:** Christine M Mueller, Larissa A Korde, Mary L McMaster, June A Peters, Gennady Bratslavsky, Rissah J Watkins, Alex Ling, Christian P Kratz, Eric A Wulfsberg, Philip S Rosenberg, Mark H Greene

**Affiliations:** 1 Clinical Genetics Branch, Division of Cancer Epidemiology and Genetics, National Cancer Institute, National Institutes of Health, Bethesda, MD, USA; 2 Division of Medical Oncology, University of Washington/Seattle Cancer Care Alliance, Seattle, WA, USA; 3 Genetic Epidemiology Branch, Division of Cancer Epidemiology and Genetics, National Cancer Institute, National Institutes of Health, Bethesda, MD, USA; 4 Urologic Oncology Branch, National Cancer Institute, National Institutes of Health, Center for Cancer Research, Bethesda, MD, USA; 5 Westat, Inc, 6116 Executive Boulevard, Suite 400, Rockville, MD, USA; 6 Diagnostic Radiology Department, National Institutes of Health, Warren G. Magnuson Clinical Center, Bethesda, MD, USA; 7 Division of Human Genetic, Department of Pediatrics, University of Maryland School of Medicine, Baltimore, MD 21201, USA; 8 Biostatistics Branch, Division of Cancer Epidemiology and Genetics, National Cancer Institute, National Institutes of Health, Bethesda, MD, USA

**Keywords:** Familial testicular cancer, Dysmorphology, Developmental anomalies

## Abstract

**Background:**

Testicular germ cell tumor (TGCT) is the most common malignancy in young men. Familial clustering, epidemiologic evidence of increased risk with family or personal history, and the association of TGCT with genitourinary (GU) tract anomalies have suggested an underlying genetic predisposition. Linkage data have not identified a rare, highly-penetrant, single gene in familial TGCT (FTGCT) cases. Based on its association with congenital GU tract anomalies and suggestions that there is an intrauterine origin to TGCT, we hypothesized the existence of unrecognized dysmorphic features in FTGCT.

**Methods:**

We evaluated 38 FTGCT individuals and 41 first-degree relatives from 22 multiple-case families with detailed dysmorphology examinations, physician-based medical history and physical examination, laboratory testing, and genitourinary imaging studies.

**Results:**

The prevalence of major abnormalities and minor variants did not significantly differ between either FTGCT individuals or their first-degree relatives when compared with normal population controls, except for tall stature, macrocephaly, flat midface, and retro-/micrognathia. However, these four traits were not manifest as a constellation of features in any one individual or family. We did detect an excess prevalence of the genitourinary anomalies cryptorchidism and congenital inguinal hernia in our population, as previously described in sporadic TGCT, but no congenital renal, retroperitoneal or mediastinal anomalies were detected.

**Conclusions:**

Overall, our study did not identify a constellation of dysmorphic features in FTGCT individuals, which is consistent with results of genetic studies suggesting that multiple low-penetrance genes are likely responsible for FTGCT susceptibility.

## Background

Although testicular germ cell tumors (TGCT) account for only 1% of malignancies in males, it is the most common malignancy among men aged 20–35 years [1]. A familial predisposition has been well documented; sons of men with TGCT have a 4- to 6-fold increased risk compared with the general population and brothers of affected siblings have an 8- to 10-fold increased risk [2,3]. The higher relative risk of TGCT among siblings than among fathers/sons suggests both genetic heterogeneity and environmental influences, including possible intrauterine exposures. Cases of ovarian germ cell tumors have also been reported in Familial TGCT (FTGCT) kindreds [4]. FTGCT has not been definitively linked to any known hereditary cancer syndrome, and unraveling the genetic basis through traditional linkage studies has been difficult, in part because families with many affected individuals are exceedingly rare [5,6].

Autosomal dominant, autosomal recessive and X-linked patterns of inheritance are seen in FTGCT families, suggesting considerable genetic heterogeneity. An autosomal recessive model has provided the best data fit in the two segregation analyses performed to date, and several genomic regions of interest have been identified in linkage analyses, but no high-penetrance susceptibility genes have yet been identified [7-11]. We reported no disease-associated germline cytogenetic abnormalities in either the 28 FTGCT men we studied by high-resolution chromosome analysis and spectral karyotyping, or 17 previously-reported FTGCT men [12]. A Y-chromosome deletion (gr/gr) has been identified as conferring 2- and 3-fold increases in risk of sporadic and familial testicular cancers, respectively, in a small percentage of men, and reports have identified germline variants in *PDE11A* and *DND1* as candidate modifiers of familial testicular cancer risk [13-15]. Three genomewide association studies (GWAS) of unselected testicular cancer patients have identified single nucleotide polymorphisms that are strongly associated with TGCT risk [16-19]. Kratz *et al*., confirmed findings of *BAK1*, *DMRT1*, *TERT*-*CLPTM1L*, and *KITLG* variants in familial and bilateral cases of TGCT [20]. A recent meta-analysis of pooled GWAS data has identified 5 additional candidate susceptibility loci, one (*UCK2*) previously identified as of possible (but not statistically significant) interest in a prior GWAS, as well as 4 novel loci: *HGPDS*, *MAD1L1*, *RFWD3 and 17q22.2*[21,22]. Most recently, *DAZL* and *PRDM14* have been implicated as well [23]. In addition, a strong correlation between LINE-1 methylation levels among affected father-son pairs suggested possible transgenerational inheritance of an epigenetic event that may be associated with disease risk [24]. Overall, these data suggest that a single major locus may not account for the majority of the familial aggregation of TGCT, but rather that multiple low-penetrance susceptibility loci acting in concert may be responsible for the genetic component of TGCT etiology.

Several additional risk factors have been described, including cryptorchidism, inguinal hernia, infertility and contralateral testicular cancer [25,26]. Previous case reports have linked TGCT with diverse congenital abnormalities including retroperitoneal anomalies (*e.g*. renal agenesis, duplicated collecting system, retro-aortic renal vein) and supernumerary nipples [27,28]. It has been postulated that TGCT stems from abnormal gonadal development during embryogenesis, and may be part of a “testicular dysgenesis syndrome,” characterized by urogenital abnormalities, subfertility, testicular microlithiasis, and testicular carcinoma *in situ*, and hypothesized as related to both environmental and genetic risk factors [29,30]. Furthermore, GCTs have been reported in a number of individuals with hereditary disorders or constitutional chromosome abnormalities, many of which also include other urogenital abnormalities [26,31].

Detailed physical examination for evaluation of minor morphologic abnormalities in conjunction with detection of major congenital anomalies is a major tool for characterizing syndromes in clinical genetics and can be helpful in guiding molecular studies [32,33]. As part of our multidisciplinary, etiologically-focused attempt to refine the FTGCT phenotype, the putative intrauterine origin of TGCT led us to hypothesize the existence of unrecognized dysmorphic features or congenital anomalies in this syndrome [34]. Since no one had previously performed a systematic dysmorphology evaluation of FTGCT family members, we comprehensively evaluated men with FTGCT and their 1st-degree relatives in search of an excess of mild errors of morphogenesis and congenital anomalies to further define the FTGCT phenotype and to provide new insights into the genetic and/or environmental etiology of TGCT.

## Methods

The objectives of the Clinical Genetics Branch Multidisciplinary Etiologic Study of Familial Testicular Cancer (NCI Protocol 02-C-0178; NCT-00039598; http://familial-testicular-cancer.cancer.gov) included identifying possible testicular cancer susceptibility genes and characterizing more precisely the clinical phenotype of individuals with FTGCT [34]. In brief, families containing ≥2 family members with documented germ cell tumors were recruited. Families with a single male displaying bilateral TGCT were also included, because of its known association with FTGCT. To date, we have enrolled 665 members (including 203 FTGCT individuals) of 127 eligible families. Willing study participants were invited to the NIH Warren G. Magnuson Clinical Center for a research evaluation; 155 members (including 61 FTGCT individuals) of 37 families have elected to attend. For the current analysis, the first 38 TGCT cases and 41 first-degree relatives from 22 multiple-case families were studied with detailed dysmorphology examinations, physician-based medical history and physical examination, laboratory testing, ultrasound imaging of the testes and ovaries, computed tomography or ultrasound of the abdomen, and computed tomography of the chest. All participants completed detailed family history, medical history, and risk factor questionnaires. This study was reviewed and approved by the National Cancer Institute (NCI) Institutional Review Board (NCI Protocol 02-C-0178), and all participants provided written informed consent.

All dysmorphology examinations were performed by one of three trained clinical geneticists (EAW, MLM, CMM). A standardized data collection instrument was developed by two of these geneticists (EAW, MLM) to insure complete, systematic assessment of features. Diagnostic criteria were applied as described by Aase and Merks [35,32]. In the case of paired organs, no distinction was made between unilateral and bilateral occurrence. Height, weight, head circumference, inner and outer canthal distance, inter-pupillary distance, and hand lengths were measured with calipers and tape measure, and findings were compared with normal standards [36]. Clinical photographs of all subject’s faces were obtained and reviewed by a single examiner (CMM) during data analysis, and no features were scored differently than previous examiners. We compared the prevalence of 11 major and 54 minor anomalies in individuals with FTGCT, their 1st-degree relatives, and those reported in 923 white school age children [37]. Fisher’s Exact test was used for statistical comparisons, with a two-tailed p-value of <0.05 considered statistically significant. All statistical analyses were conducted using SPSS 15.0. Of note, retro-/micrognathia in the 923 school age children included individuals with retrognathia and micrognathia occurring separately or in combination.

## Results

The study sample included 38 men with FTGCT and 41 of their unaffected first-degree relatives (21 males, 29 females) from 22 multiple-case white TGCT families (Table 1). Median age at TGCT diagnosis was 31 years (range: 15–56), and the usual mix of seminomatous and non-seminomatous tumors among the families was observed. Multiple patterns of inheritance were observed, including nine families with FTGCT brothers, 9 with FTGCT father-sons, 2 with FTGCT cousins, 1 with FTGCT uncle-nephew, and 1 bilateral FTGCT individual. We examined 41 unaffected first-degree relatives, 21 males and 20 females. Seven families did not have a first-degree relative available for examination. The median ages of FTGCT men, unaffected men, and women at the time of study were 40 (21–72), 31 (14–68), and 47 (15–67), respectively.

**Table 1:** Composition of FTGCT families examined

**Family**	**Relationship among FTGCT family members**	**FTGCT family members examined**	**Unaffected 1st degree family members examined**
1	Father/Son	1	0
2	Father/Son	1	1
3	Father/Son	2	0
4	Father/Son	2	0
5	Father/Son	2	4
6	Father/Son	2	7
7	Father/Son	2	2
8	Father/Son	1	2
9	Father/Son	2	0
10	Siblings	2	3
11	Siblings	2	2
12	Siblings	1	0
13	Siblings	2	3
14	Siblings	1	0
15	Siblings	3	0
16	Siblings	1	3
17	Siblings	2	4
18	Siblings	3	1
19	Cousins	2	1
20	Cousins	2	3
21	Uncle/Nephew	1	2
22	Bilateral	1	3

The prevalence of major abnormalities and minor variants did not significantly differ between either men with FTGCT or their first-degree relatives when compared with the normal population controls, except for tall stature, macrocephaly, flat midface, and retro-/micrognathia (Table 2). One mother had a previous diagnosis of Holt Oram syndrome (OMIM 142900), but otherwise no major abnormalities of the extremities or skeletal dysplasias were found in our cases or their relatives, so they are excluded from Table 2.

**Table 2:** **Prevalence of congenital abnormalities in FTGCT individuals**, **first degree relatives**, **and normal school age children** (%)

	**FTGCT (N = 38)**	**1st degree relatives (N = 41)**	**Normal population (N = 923)**
***Major abnormality***			
Short stature (proportionate)	0	0	2.0
Cleft lip	0	0	0
Cleft palate	0	0	0
Ear tags	0	0	0.3
Ear pits	2.6	0	1.1
Webbed neck	0	0	0
Supernumerary nipples	2.6	2.4	2.8
Talipes equinovarus	0	0	0
2,3 toe syndactyly	0	2.4	0.4
Joint hypermobility	0	0	10.3
Joint contractures	0	0	0
***Minor variant***			
Tall stature (proportionate) *p* = *0.05*^††^	7.9	2.4	2.0
Macrocephaly^*^p = *0.01*^††^	11.1	4.9	2.0
Microcephaly	2.6	0	2.0
Abnormal hair whorl	0	0	0.1
Widow’s peak	5.3	2.4	6.7
Coarse face	2.6	2.4	0.5
Prominent forehead	0	0	2.4
Facial asymmetry *p* < *0.01*^††††^	5.3	9.8	1.6
Flat mid-face *p* < *0.01*^†††^	23.7	2.4	1.0
Hypertelorism	0	0	2.0
Hypotelorism	0	0	2.0
Telecanthus	0	0	0.4
Upslanting palpebral fissures	2.6	2.4	3.9
Downslanting palpebral fissures	5.3	2.4	0.8
Epicanthal folds	2.6	0	3.5
Ptosis	0	2.4	4.4
Broad nose	0	2.4	1.1
Short nose	2.6	0	9.3
Broad nasal tip	5.3	7.3	2.6
Anteverted nares	0	0	4.2
Hypoplastic alae nasae	0	0	0.7
Smooth philtrum	0	0	5.3
Prominent philtrum	5.3	0	1.8
Prominent upper jaw	0	0	2.1
Retro-/micrognathia *p* = *0.04*^††^	7.9	4.9	1.7
Prominent lower jaw	0	0	0.3
Pointed chin	2.6	2.4	0.7
High-arched palate	2.6	4.9	6.2
Bifid uvula	0	0	0
Extra frenulae	0	0	0
Abnormally shaped teeth	2.6	0	1.6
Lowset ears	0	0	0.5
Posteriorly rotated ears	2.6	4.9	1.4
Overfolded helices	7.9	4.9	4.1
Darwinian tubercle	5.3	0	4.6
Ear lobe crease	2.6	2.4	0
Attached ear lobes	2.6	2.4	12.8
Pectus excavatum	5.3	7.3	2.3
Pectus carinatum	0	2.4	0.3
Gynecomastia	0	0	0.1
Absent/hypoplastic nipples	0	0	0
Wide-spaced nipples	2.6	0	0.4
Inverted nipples	2.6	0	4.1
Bridged palmar crease	0	2.4	2.7
Single transverse crease	0	2.4	2.3
Sydney crease	0	0	0.3
Clinodactyly	0	0	3.6
Partial 2,3 toe syndactyly	0	2.4	0.3
Hammer toes	2.6	0	0.2
2nd toe longer than 1st	7.9	7.3	3.1
Pes planus	0	2.4	2.6
Café-au-lait spots	13.2	12.2	13.5
Hemangiomas	2.6	2.4	0.7
Port wine stain	2.6	0	0.2

^††^Between FTGCT individuals and normal population.

^†††^Between FTGCT individuals and normal population and FTGCT individuals and relatives.

^††††^Between relatives and normal population.

*36 FTGCT individuals measured.

The prevalence of tall stature, macrocephaly and retro-micrognathia was significantly greater between men with FTGCT and the normal population, but not between men with FTGCT and their 1st- degree relatives. All individuals with macrocephaly were only mildly affected, and there was no evidence of familial aggregation for this trait. When head circumference was plotted against height, as suggested by Bushby *et al*., only one affected male remained mildly macrocephalic, and this feature was no longer statistically significant [38]. Of note, retro-/micrognathia included individuals with retrognathia and micrognathia occurring separately or in combination [37].

Flat mid-face was statistically significant between FTGCT individuals and the general population and their 1st-degree relatives. There was no familial aggregation among the 9 FTGCT individuals and 1 first-degree relative with flat mid-face. Facial asymmetry was seen more frequently in unaffected relatives than either their affected relatives or the normal population, and there was no evidence of familial aggregation. These 4 traits were not manifest as a constellation of features in any one individual or family.

Table 3 summarizes the prevalence of congenital genitourinary tract abnormalities in FTGCT males *vs*. unaffected males. By history, cryptorchidism was more frequent among cases than among unaffected men, 13% *vs*. 5% and, when compared with the highest estimates in the general population, 4%, was statistically significant [39,40]. The prevalence of congenital inguinal hernia was similar in FTGCT males vs. unaffected family members (18.4% *vs*. 19%), statistically significantly higher than the occurrence in the general population, 5% [41]. One FTGCT male and one unaffected male from a different family had bilateral duplicated renal collecting systems (2.6% vs. 2.4%). This is one of the most common renal abnormalities, and occurs in approximately 1% of the population [42,43]. No other congenital renal, retroperitoneal or mediastinal abnormalities were found in our thoraco-abdominal imaging studies.

**Table 3:** **Prevalence of congenital genitourinary tract abnormalities** (%)

**Condition**	**FTGCT individuals (N = 38)**	**1st Degree unaffected male relatives (N = 41)**	**Normal population**
Cryptorchidism* *p* < *0.05*^††^	13.2	4.8	4.0
Congenital inguinal hernia* *p* < *0.01*^†††^	18.4	19.0	5.0
Duplicated collecting system	2.6	2.4	1.0

*Includes only the 21 male 1st Degree relatives.

^††^Between FTGCT individuals and normal population and FTGCT individuals and relatives.

^†††^Between FTGCT individuals and normal population and relatives and normal population.

## Discussion

Our study provides the largest, most comprehensive descriptive analysis of formal dysmorphology evaluations in individuals from familial TGCT kindred. The current analysis is part of the only systematic, multidisciplinary etiologic study of extended multiple-case TGCT families being conducted in the world, and thus represents a unique opportunity to assess an important hypothesis: given the likely intra-uterine origins of testicular neoplasia, is there a dysmorphic and/or a congenital anomaly component to the FTGCT syndrome phenotype? We compared the prevalence of 11 major and 54 minor anomalies in 38 males from FTGCT families with those of 923 normal children. To assess familial aggregation and potential unidentified carriers of as yet unknown testicular cancer susceptibility genes, we also examined 41 available, unaffected 1st-degree relatives. No notable pattern of dysmorphic features was detected between FTGCT individuals and either their relatives or population controls, nor was there evidence of excess renal, retroperitoneal or mediastinal congenital anomalies.

Compared with the normal population, FTGCT men were more likely to have tall stature, macrocephaly, and retro- and/or micro-gnathia. They were also more likely to have these traits than their unaffected relatives, but these differences were not statistically significant. It is theoretically possible that clinically unaffected relatives displaying one or more of these traits are unidentified carriers of a familial TGCT phenotype. However, there was no familial aggregation of macrocephaly or retro-and/or micro-gnathia and no single individual manifested a constellation of these traits, making it difficult to identify a pattern of morphologic features that might be uniquely associated with familial TGCT. Furthermore, as familial aggregation of tall stature may occur in the general population, we also performed the analysis by excluding the one FTGCT individual and his daughter who were unusually tall, and tall stature was no longer statistically significant. The validity of the statistical significance of the prevalence of retro-micrognathia is difficult to discern, as the only available control data were comprised of retrognathia and micrognathia combined; typically, these are considered two distinct features [37].

Flat mid-face was significantly more common in affected males than either their relatives or the normal controls, suggesting that it might be a trait that is part of a familial TGCT phenotype. In addition, facial asymmetry was more common in unaffected relatives than the normal population or affected males. However, these traits are subjectively defined by the examiner and also may vary in any one individual depending on other physical features, such as weight and age at examination.

Previous reports have linked cases of TGCT with congenital abnormalities including retroperitoneal urinary anomalies and supernumerary nipples [27,28]. There were no differences in the prevalence of supernumerary nipples in any of the patient subsets. We also did not detect an increased frequency of renal, retroperitoneal or mediastinal abnormalities on thoraco-abdominal imaging in our families. We observed a higher prevalence of cryptorchidism among FTGCT cases than among unaffected relatives and the general population, as has been repeatedly described in sporadic TGCT. In addition, we observed a higher prevalence of congenital inguinal hernia in FTGCT cases and their unaffected relatives compared with the general population. FTGCT cases and their relatives with congenital genitourinary tract anomalies were not more likely to have the anomalies listed in Table 2.

The major strength of this study is that all participants underwent a comprehensive clinical evaluation by a small group of dysmorphologists who employed a standardized data collection form. In addition, study participants systematically provided detailed medical and family history information. This study is the first to examine men with FTGCT and their family members in sufficient detail to determine if there is a pattern of morphologic features common to this disorder.

The findings in our study are limited by the small sample sizes for both the affected males and their first-degree relatives, which results in minimal statistical power to detect differences between study sub-groups. In addition, our ability to detect differences in the subjective evaluation between examiners was limited by not having each participant evaluated by multiple examiners or having the photographs of participants evaluated by CMM scored by the other examiners. Furthermore, we might have been able to identify a larger number of abnormalities if we had access to all first-degree relatives of FTGCT individuals. Larger studies would be required to further characterize these traits in individuals with FTGCT but, pragmatically, it is unlikely that such data will be forthcoming in the foreseeable future. Comparing anomaly prevalence in our subjects to rates from a large, literature-based normal population may have been sub-optimal when evaluating traits that are subjectively defined by different examiners. In addition, the control population consisted of school age children from the Netherlands compared with our adult population from the United States; however, we believe that these white populations should be similar in morphological features and that minor differences that more commonly occur in adults compared with children (such as male pattern baldness or striae) were not included in our analysis. Finally, our study population may not be representative of all FTGCT families, since it was comprised of research volunteers who were willing to travel to the NIH Clinical Center for an in-person evaluation.

## Conclusions

Our study did not identify a constellation of dysmorphic or congenital anomalies in affected males from multiple-case TGCT families or their unaffected close relatives. Based on the data that we were able to collect from this rare and unique population, it appears that this strategy would not be helpful in guiding ongoing molecular and etiologic studies. Furthermore, our findings are consistent with results of genetic studies that have been done to date, in that a single gene does not appear to account for the majority of the familial aggregation of TGCT; overall, the descriptive epidemiology of familial and sporadic testicular cancer are remarkably similar.

Our data provide further support for the hypothesis that multiple, common low-penetrance genetic variants are more likely responsible for FTGCT susceptibility rather than a rare, highly-penetrant gene of major effect.

## Competing interests

The authors declare that they have no competing interests.

## Authors’ contributions

CMM, LAK, MLM, JAP, RJW, EAW, and MHG made substantial contributions to the conception and design of the study. CMM, MLM, and EAW designed the dysmorphology data collection and performed dysmorphology examinations. GB was responsible for genitourinary examination and AL and GB were responsible for radiologic examination review and contributed to the conception and design of the study. CMM performed data collection and statistical analysis. CMM, PSR, CPK, and MHG interpreted data and drafted the manuscript. All authors read and approved the final manuscript.
